# Carbon Nanofiber-Ionic Liquid Nanocomposite Modified Aptasensors Developed for Electrochemical Investigation of Interaction of Aptamer/Aptamer–Antisense Pair with Activated Protein C

**DOI:** 10.3390/bios13040458

**Published:** 2023-04-04

**Authors:** Meltem Maral, Arzum Erdem

**Affiliations:** 1 Department of Material Science and Engineering, The Institute of Natural and Applied Sciences, Ege University, Bornova, 35100 Izmir, Turkey; 2 Analytical Chemistry Department, Faculty of Pharmacy, Ege University, Bornova, 35100 Izmir, Turkey

**Keywords:** carbon nanofibers, ionic liquid, activated protein C, electrochemical impedance spectroscopy, aptasensors

## Abstract

Selective and sensitive detection of human activated protein C (APC) was performed herein by using carbon nanofiber (CNF) and ionic liquid (IL) composite modified pencil graphite electrode (PGE) and electrochemical impedance spectroscopy (EIS) technique. A carbon nanomaterial-based electrochemical aptasensor was designed and implemented for the first time in this study for the solution-phase interaction of DNA-Apt with its cognate protein APC as well as APC inhibitor aptamer–antidote pair. The applicability of this assay developed for the determination of APC in fetal bovine serum (FBS) and its selectivity against different proteins (protein C, thrombin, bovine serum albumin) was also examined. CNF-IL modified aptasensor specific to APC provided the detection limit as 0.23 μg/mL (equal to 3.83 nM) in buffer medium and 0.11 μg/mL (equal to 1.83 nM) in FBS. The duration of the proposed assay from the point of electrode modification to the detection of APC was completed within only 55 min.

## 1. Introduction

Nanomaterial-modified surfaces provided an excellent medium for developing nucleic acid biosensors that enables more selective and sensitive analysis [1]. Carbon-based nanomaterials have become potential candidates for electrochemical biosensors due to their advantages such as a high surface/volume ratio, good chemical stability, and biocompatibility [2,3,4].

Carbon nanofibers are the advanced type of carbon nanomaterials that have similar conductivity and stability as carbon nanotubes. The main difference between carbon nanofibers and carbon nanotubes is the piling of graphene plates of various figures, which provides more edge area on the outer wall of carbon nanofibers than carbon nanotubes. Thus they can facilitate the electron transfer of electroactive analytes. The unmatched chemical and physical properties allowed carbon nanofibers to be used as electrode materials and immobilization substrates [5]. As carbon nanofibers have stand-alone applications in electrochemical biosensors, electrochemical biosensors based on composites formed with conductive polymers and other conductive materials have applications for sensitive and selective determination of analytes such as proteins, nucleic acid, and drugs [6,7,8].

Ionic liquids (IL) are molten salts that have a melting point near or lower than room temperature [9]. IL offer many advantages such as high chemical and thermal stability, negligible vapour pressure, high ionic conductivity, wide electrochemical windows, low toxicity, and the ability to dissolve a wide variety of organic and inorganic compounds [10]. Ionic liquids (IL) have replaced organic solvents because of their attractive properties. The major application of IL in the biosensing area is related to their implementation in environmental electrochemical (bio)sensors due to their specifications as good conductors as well as binders [11,12,13,14].

Owing to the extensive electrochemical applications of ionic liquids, they can be used as electrode material. They have also been incorporated into traditional materials such as chitosan, carbon nanotubes, polymer, and Nafion to improve conductivity and promote electron transfer [15]. By dispersing the CNFs in the IL, the conductivity of the CNFs is greatly increased. For electrochemical detection systems, the electrochemical performance of the CNF-IL composite material, which is formed by combining the advantages of IL and CNF, provides a remarkable increase. For this reason, the number of ionic liquid/nanomaterial composite applications in electrochemical analysis has been recently increasing day by day [5,16,17,18,19,20,21,22,23].

Aptamers are DNA or RNA-based synthetic nucleic acids that are capable of defining organic or inorganic targets such as drugs, toxins, amino acids, proteins, and living cells [24,25,26,27,28,29]. In the current studies in the literature, they show an increasing interest in biosensor applications as potential therapeutic agents or recognition elements, that are also used to design sensitive and selective biosensors specific to the target [8]. In the mentioned uses, the importance of characterizing the interaction between the target and the aptamer in the solution phase or on the electrode surface has increased [30,31]. Aptamers offer some unsurpassed properties such as high binding affinity, better stabilization, and longer shelf life compared to antibodies and they can recycle and release their targets.

A variety of aptamer-based biosensors has been developed in combination with different detection techniques. Electrochemical impedance spectroscopy (EIS), which is the one of electrochemical techniques, is a simple and sensitive technique. EIS is widely used in the development of aptasensors [29,30,31]. In contrast, to detect proteins by using EIS technique, traditional techniques have been developed for protein analysis such as high-performance liquid chromatography (HPLC), gas chromatography (GC), and enzyme-linked immunosorbent assays (ELISAs) which are time-consuming methods with logistics requirements as well as requiring specialized personnel and high-cost devices [32,33,34,35,36].

Protein C (PC) is a zymogen protein that functions in the anticoagulant mechanism to prevent excessive coagulation, and decreased protein C levels are related to neonatal purpura fulminans and venous thrombosis. Activated protein C (APC) is a serine protease produced from inactive precursor protein C. Activated protein C is the key enzyme of the protein C pathway and acts as a strong inherent anticoagulant by inactivating factors Va and VIIIa [37]. Uncontrolled inflammation and coagulation are two hallmarks of sepsis. After the discovery of a relationship between the decrease in endogenous protein C and APC, the recombinant human APC (rhAPC) was developed for the treatment of patients with sepsis. APC binds to protein S and inhibits factor Va and factor VIIIa to prevent any more thrombin formation, thereby regulating blood clotting [38,39].

The therapeutic use of APC in severe sepsis is an important inflammatory component that has been evaluated. In addition, it is also applied for the treatment of diseases such as obesity, pneumonia, diffuse intravascular coagulation, sepsis, chronic renal failure, etc. [40,41,42,43,44]. Activated protein C is a serious organizer of thrombin creation and this property prevents thrombosis formation. Excessive APC formation increases bleeding risk, like traumatic coagulopathy, and therefore pharmacological inhibition of activated protein C activity might increase blood coagulation in some clinical cases. The inhibition of APC will require more time for tenase and prothrombinase complexes to produce thrombin [45]. Preclinical studies are still ongoing and encouraging conclusions have been reported for distinct molecules such as antibodies and aptamers for activated protein C inhibition and their possible use as novel therapeutic strategies in the treatment of trauma-induced coagulopathy (TIC) and haemophilia [45,46,47,48,49].

Various assays are available to measure the level of APC [50]. Most studies to measure the level of activated protein C are based on studies in which activated protein C is detected using a specific monoclonal (mAb) or polyclonal antibody (polAb), as well as enzyme detection assays (ECA), in which the amidolytic activity of activated protein C is measured using a specific chromogenic substrate [51,52,53,54,55]. Another method evaluates the activation peptide released into protein C activation [56]. Some of these methods based on sandwich ELISA performed activated protein C detection by using two antibodies, a monoclonal anti-protein C antibody and an anti-protein C inhibitor (PCI) antibody [57,58,59]. In recent years, other studies related to activated protein C analysis have focused on the development of aptamer-based biosensors [60,61,62,63,64,65]. These methods present some differences in some cases such as the amount of sample required for analysis, the detection time as well as the detection limit.

Protein C (PC), an anticoagulant protein, is the principal agent responsible for preventing blood clots and thrombosis in the human coagulation system. It is naturally present in human blood at a concentration of 4 μg/mL. As a zymogen, PC is present in the plasma and is activated by specific cleavage by thrombin bound to thrombomodulin located on the endothelial cell membranes. The conversion of PC to its active form, APC, is catalyzed by thrombin in the presence of thrombomodulin, which is a key step in the PC pathway. APC, which is produced by the activation of PC, plays a crucial role as an enzyme in the anticoagulant and antithrombotic mechanisms of the human body [28,37,60,64]. Therefore, APC levels are mainly important in pathological conditions (e.g., sepsis, and coagulation disorders) and may need to be determined sensitively and selectively in these conditions [51].

EIS has been most commonly used in the development of electrochemical aptasensors that have low impedance due to charge transfer between the electrode surface and redox probe. After the aptamer binds to its target analyte, high impedance is observed because charge transfer is prevented at the electrode surface. This strategy has been frequently used in order to identify the biointeractions of many target molecules [66,67,68,69]. In particular, EIS-based biosensors are well suited for detecting binding events occurring at the transducer surface. Thus, very small changes before/after the biointeraction process can be detected easily and quickly via EIS-based biosensors at the electrode surface [70,71]. Therefore, it is very necessary to improve its selectivity by replacing the electrode surface with a specific substance that can interact specifically with the analytes [23,68]. In one of our earlier studies [61], dendrimer modified impedimetric aptasensor was designed and applied to detect the interaction of APC-specific DNA aptamer (DNA-Apt) with its target protein, activated protein C. Koyun et al. [28], developed a highly sensitive and selective label-free aptasensor for the specific detection of human activated protein C by surface plasmon resonance method. In another work published in 2019 by Ghalehno et al. [65], a sensitive and selective voltammetric aptasensor for the determination of activated protein C was reported using methylene blue as a redox indicator.

Hamedani et al. [47] investigated the effect of the APC-specific DNA aptamer HS02-52G on the restoration of blood coagulation in plasma and whole blood coagulation models affected by APC. In their study, the most effective of the HS02-52G neutralizing antisense molecules was investigated. According to their results, it was reported that the antidote pair formed in the presence of the antisense oligonucleotide molecule AD22, which was conjugated with HS02-52G and acted as an effective APC inhibitor. Thus, the aptamer-antisense-based antidote couple resulted a new potential biotechnology product, that has been proposed as a rescue treatment option for potentially acute APC-related bleeding complications, for example in traumatic coagulopathy [47,72].

As far as we know, there is no report on the development of solution phase aptasensor specific to APC and its application to APC analysis using CNF-IL composite modified electrodes in combination with EIS technique. Under this scope, the carbon nanomaterial-based electrochemical aptasensor was designed and applied for the first time for the solution-phase interaction of APC and APC-specific DNA aptamer as well as APC inhibitor; aptamer–antidote pair. The experimental parameters were optimized for the solution-phase interaction of aptamer with APC in different concentrations, the interaction of APC with the antidote pair (Apt/AD22) in the absence/presence of the DNA-Apt and its corresponding antisense oligonucleotide (AD22). Additionally, the selectivity of APC aptasensor was explored in the presence of different proteins (protein C, thrombin, bovine serum albumin). The applicability of the impedimetric aptasensor for APC detection was also explored in fetal bovine serum (FBS).

## 2. Experimental Section

### 2.1. Apparatus

All measurements were performed using a three-electrode system and also in a faraday cage by AUTOLAB-302 (Eco Chemie, Utrecht, The Netherlands) with the NOVA 1.11 software and the FRA module. For electrochemical measurements, cyclic voltammetry (CV) and electrochemical impedance spectroscopy (EIS) were used. All measurements were carried out at room temperature. The three-electrode system consisted of a pencil graphite electrode (PGE) as the working electrode, a platinum wire as an auxiliary electrode, and Ag/AgCl/3M KCl as the reference electrode (BAS, Model RE-5B, W. Lafayette, IN, USA).

#### 2.1.1. Electrode Preparation

A Tombow pencil tip was used as the graphite lead holder as the electrode. Electrochemical treatment was applied to each electrode tip that was used for electrochemical measurements as reported in earlier studies [60,61,62,73,74].

#### 2.1.2. Chemicals

All oligonucleotides used in this study were purchased from Ella Biotech GmbH (Fürstenfeldbruck, Germany). Carbon nanofiber (CNF) and 1-butyl-3-methylimidazolium hexafluorophosphate (IL) were acquired from Sigma-Aldrich. Activated protein C (APC) was purchased as lyophilized from Haematologic Technologies (Essex Junction, VT, USA). Thrombin (THR), bovine serum albumin (BSA), and protein C (PC) were purchased as lyophilized from Sigma-Aldrich (Munich, Germany). Fetal bovine serum (FBS) was also purchased from Sigma-Aldrich (Germany).

The stock solutions of proteins were prepared by dissolving in fresh ultrapure triple-solutions. Then, the diluted solutions of proteins were prepared in 50 mM phosphate buffer solution containing 20 mM NaCl (PBS, pH 7.40).

The base sequences of DNA-Apt and AD22 are given below:

APC specific DNA-Apt: 5′-NH_2_-GCC TCC TAA CTG AGC TGT ACT CGA CTT ATC CCG GAT GGG GCT CTT AGG AGG C-3′.

Antisense oligonucleotide (AD22): 5′-ATC CGG GAT AAG TCG AGT ACA G-3′

#### 2.1.3. Preparation of DNA-Apt and AD22

DNA-Apt was prepared according to the information given in the literature [60,61,62,63]. 500 µg/mL DNA-Apt was prepared in ultrapure water and kept frozen. More diluted DNA-Apt solutions were prepared in PBS (pH 7.4). A total of 1000 µg/mL AD22 was prepared in ultrapure water and kept frozen. More diluted AD22 solutions were prepared in PBS (pH 7.4).

#### 2.1.4. Preparation of APC, PC, BSA, or THR Solution

The APC solution was prepared using ultrapure water and kept frozen. More diluted APC solutions were prepared in PBS (pH 7.4). Other chemicals were analytical reagent grade and were purchased from Sigma (St. Louis, MO, USA) and Merck (Rahway, NJ, USA). Ultra-pure and deionized water is used in all solutions.

#### 2.1.5. The Preparation of CNF-IL Dispersion

The required amount of IL was dispersed in dimethyl formamide (DMF) to prepare of 5% IL solution and it was kept for the sonication step for 5 min. The specified amount of CNF was then added to the 5% IL solution, and this mixture was then sonicated for 25 min.

### 2.2. Procedure

**Scheme 1:**
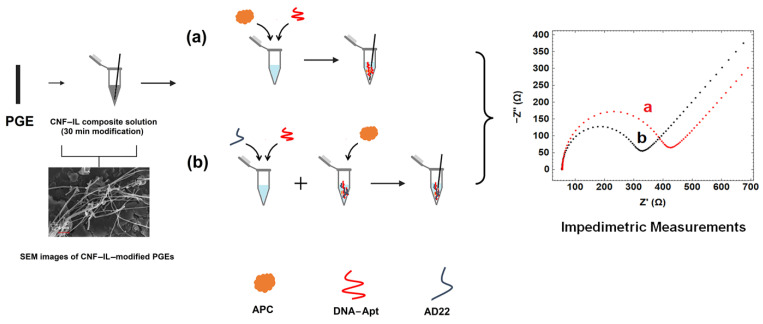
Scheme represents the experimental procedure for the development of an impedimetric aptasensor specific to APC with the representative impedimetric data obtained interaction of DNA aptamer with APC (**a**) in the absence of the AD22 antisense, (**b**) in the presence of the AD22 antisense.

The experimental procedure was carried out by the following steps (Scheme 1):

- First, the carbon nanomaterial, CNF, was dispersed in the solution of IL by sonication for 30 min.
- The obtained CNF/IL composite material was modified onto the electrode surface for 30 min.
- The interaction of DNA-Apt with APC in its various concentration and the detection of APC was investigated using the EIS method and CNF/IL modified disposable electrode (a). After the aptamer binds to its target APC, an increase in Rct value was observed since the charge transfer is prevented on the electrode surface.
- The interaction of aptamer–antisense pair (AD22), which is effective in APC inhibition, with APC was carried out. The interaction of DNA-Apt, AD22 and APC in its various concentration and the detection of APC was investigated using the EIS method and CNF/IL modified disposable electrode First, the interaction of DNA-Apt and AD22 in the solution phase was performed for 2 min, then APC was added into the medium. After that, this sample was mixed for one minute. Accordingly, a decrease in Rct value was observed in the presence of DNA-Apt-AD22 complex containing APC (b).

The experimental procedure followed for the preparation of CNF-IL-modified PGE and the impedimetric determination of APC in the absence/presence DNA-Apt and AD22 by CNF-IL/PGE is shown in Scheme 1.

#### 2.2.1. Preparation of IL-CNF/PGEs

PGEs were first electrochemically pretreated in ABS by applying +1.40 V for 30 s. In a study to examine the effect of IL percentage upon the response, various percentages of IL (2-3-5 and 7%) were used for the preparation of composite modified with CNF-IL as similarly in our previous study [6]. The CNF-IL composite solution was prepared as a mixture of IL (5%) and CNF (500 µg/mL) and the electrodes were dipped into these samples for the modification step for 30 min. Then, the CNF-IL/PGE electrodes were left to dry for 10 min.

#### 2.2.2. Preparation of Aptamer Immobilized IL-CNF Modified Electrodes and the Interaction of Aptamer with APC

Each of these electrodes was immersed into the vials containing the required amount of DNA-Apt. Then, the interaction between DNA aptamer and APC was carried out in the solution phase for a 5 min interaction time chosen in our earlier study [6]. The electrodes were then rinsed with PBS for 10 s. In each step, an impedimetric measurement was performed and the Rct value was recorded. For testing the selectivity of aptamer against other proteins THR, PC and BSA in contrast to APC, the same experimental steps were followed by using CNF-IL/PGE.

#### 2.2.3. Impedimetric Detection of Interaction of DNA Aptamer with APC in the Presence of Its Antidote

The interaction of APC with APC-specific DNA aptamer in the after/before of its antidote (AD22) was performed in different interaction times varying from 1 to 5 min. In each step, an impedimetric measurement was carried out and the change in Rct value was recorded. In the control experiment, the measurement was done individually in the presence of APC, DNA-Apt, and AD22.

#### 2.2.4. Electrochemical Measurements

The voltammetric measurements were performed by using the Cyclic voltammetry (CV) technique in 2 mM K_3_[Fe(CN)_6_]/K_4_[Fe(CN)_6_] (1:1) containing 0.01 M KCl by applying a step potential of 25 mV, a scan rate of 50 mV/s; forward scan, −0.5 to + 1.3 V; reverse scan, +1.3 to −0.5 V.

The impedimetric measurements were performed in the presence of 2.5 mM Fe(CN)_6_^3−/4−^ prepared in 0.1 M KCl. The impedance was measured in the frequency range between 100 mHz and 100 kHz at a potential of +0.23 V with a sinusoidal signal of 10 mV. The frequency interval is divided into 98 logarithmically equidistant measure points. The elements of Randles circuit values were calculated using the fitting program of NOVA (version 1.11, EcoChemie, Utrecht, The Netherlands). The equivalent circuit model (Randles circuit) is used to fit the data obtained by impedimetric measurements, which consist of charge transfer resistance (Rct), solution resistance (Rs), Warburg impedance (W), and constant phase element (C). The respective semicircle diameter corresponds to Rct. The related circuit model is used to fit the impedance data, as shown in all figures containing Nyquist diagrams that are plotted by using the data obtained by EIS measurements. All data presented in each Nyquist diagram is the fitted version of the related data.

## 3. Results and Discussion

### 3.1. Microscopic Characterization of the Electrodes by Using Scanning Electron Microscopy (SEM)

**Figure 1:**
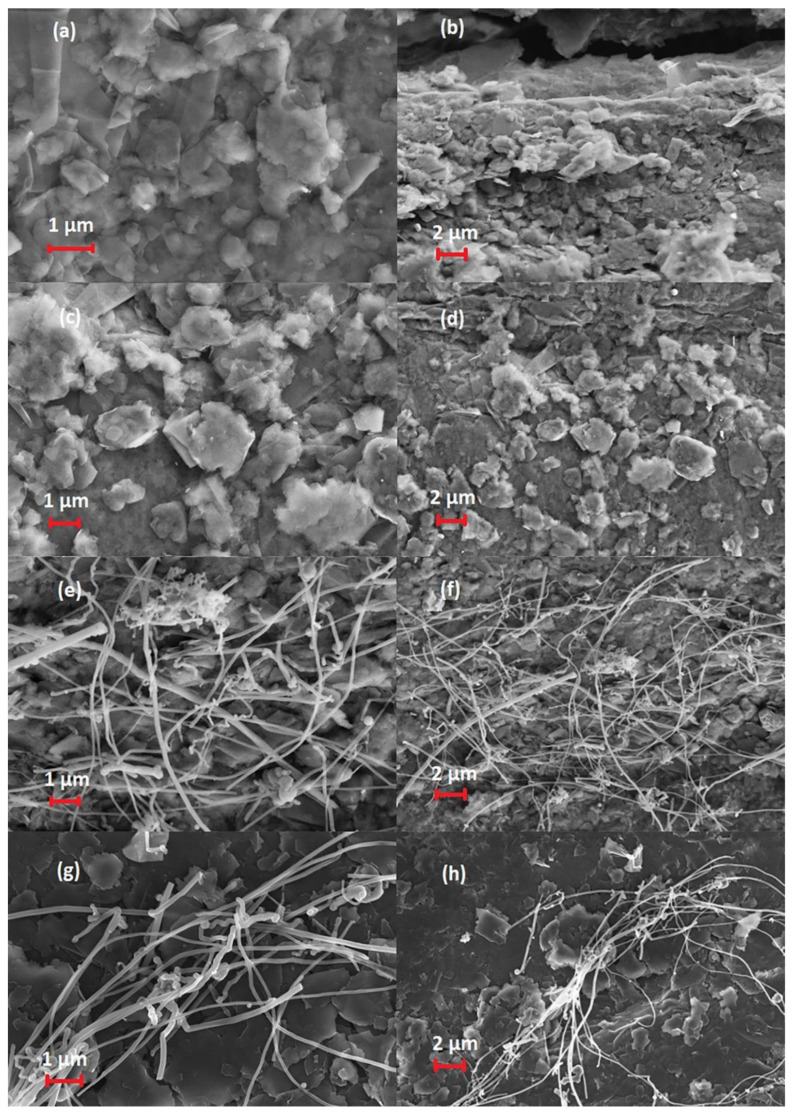
SEM images of unmodified PGE (**a**,**b**), IL/PGE (**c**,**d**), CNF/PGE (**e**,**f**) CNF-IL/PGE (**g**,**h**) using the identical acceleration voltage as 5.0 kV with resolution 1 µm and 2 µm, respectively.

Firstly, the microscopic characterization of each of the electrodes; unmodified PGE, IL/PGE, CNF/PGE, and CNF-IL/PGE was investigated in two-dimensional dimensions of 1 µm and 2 µm resolution by SEM technique and the results are shown in Figure 1.

The sheeted graphite structure of PGE was observed as shown in Figure 1a,b. After ionic liquid modification onto the surface of PGE, a less rough surface of the electrode was obtained (Figure 1c,d). After carbon nanofiber modification, nanofibers are visible at the surface of the electrode (Figure 1e,f). The presence of carbon nanofiber at the electrode surface provides a fibrous structure that is proof of the successful coating of the electrode surface with CNF-IL composite (Figure 1g,h).

The results of the EDX analysis are shown in Figures S1 and S2. Considering the chemical formulas of CNF and IL (C_8_H_15_F_6_N_2_P);

- It was observed that the carbon (%) weight level at the PGE surface gradually decreased after the modification of CNF and CNF-IL. The decrease in the carbon (%) weight level at the PGE surface was observed after CNF-IL composite modification onto the electrode surface, which is a result of the modification of the graphite layer consisting of carbon layers on the PGE surface with nanocomposite material.
- After IL modification onto the PGE surface, the presence of F and P elements was observed at the electrode surface. After modification of IL and CNF-IL onto the PGE surface, it was observed that there is a gradual decrease in the weight level of the F and P elements (%), and the highest coating at the PGE surface occurred in the presence of the CNF-IL composite modification.

### 3.2. Electrochemical Characterization of CNF/IL Modified Electrodes

**Figure 2:**
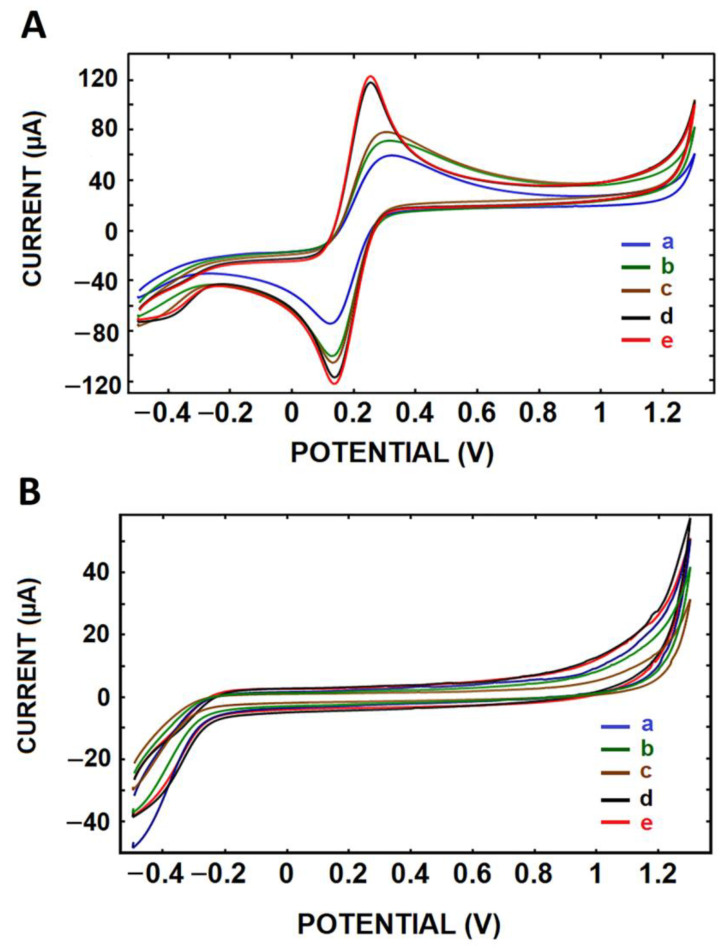
Voltammograms obtained from CV measurements (**A**) in redox prob solution (2 mM Fe(CN_6_)^3/−4^) with (a) PGE, (b) PGE (control), (c) CNF/PGE, (d) IL/PGE, (e) CNF-IL/PGE (**B**) in 0.01 M KCL solution (a) PGE, (b) PGE (control), (c) CNF/PGE, (d) IL/PGE, (e) CNF-IL/PGE (*n* = 3).

**Figure 3:**
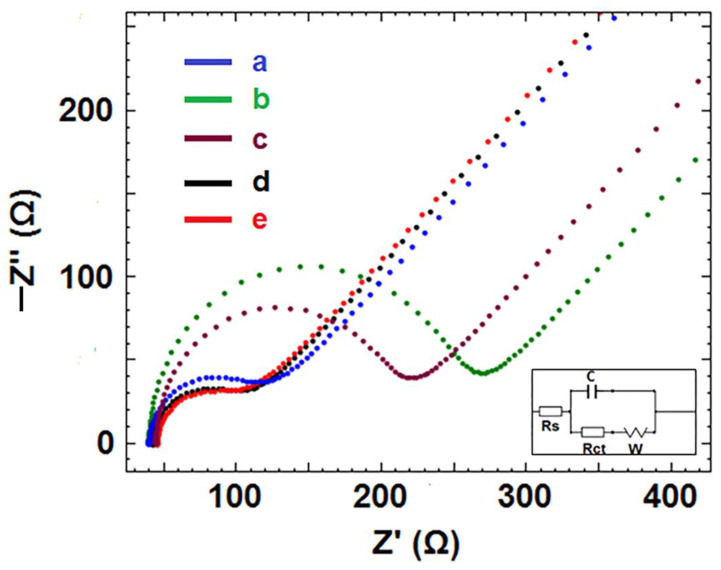
Simple equivalent circuit model in accordance with the Nyquist diagrams and impedance data obtained from the measurements performed with (a) PGE, (b) PGE (control), (c) CNF/PGE, (d) IL/PGE, (e) CNF-IL/PGE.

The electrochemical characterization by cyclic voltammetry (CV) and electrochemical impedance spectroscopy (EIS) techniques were performed before and after CNF-IL modification. Accordingly, the results were presented in Figure 2 and Figure 3 respectively. The effect of the change in the percentage of ionic liquid in the CNF-IL composite upon the aptasensor response was examined and the voltammetric results are given in Table S1. When there is an increase in the ionic liquid percentage from 2% to 5% in the CNF-IL composite, an increase was recorded at the current value with an RSD% varying from 18.13% to 2.64%. When the IL percentage was increased from 5% to 7%, a decrease in the current value was obtained. The highest and most reproducible current value was recorded by CNF-IL composite-modified electrodes with 5% ionic liquid. Thus, 5% was chosen as the optimum ionic liquid percentage.

Before and after the modification of CNF and IL onto the PGE surface, CV measurements were performed and the results were shown in Figure 2. The average values (*n* = 3) of anodic current (Ia, µA) and cathodic current (Ic, µA) with anodic/cathodic charge transfer values (mC) of the redox probe measured by CV with the calculated surface area (A, cm^2^) for the electrodes PGE, CNF/PGE, IL-PGE, and CNF-IL/PGE electrodes were shown in Table S2. In comparison to PGE, CNF/PGE, and IL/PGE, the higher current values were measured with CNF-IL/PGE electrodes. Under the optimized conditions, the effective surface area of the electrode (PGE, CNF/PGE, IL/PGE, CNF-IL/PGE) was calculated according to the Randles–Sevcik equation [75] given in Equation (1).

(1) $$I_{p}=\left(2.69\times 10^{5}\right)\;n^{3/2}{\;A\;C\;D}^{1/2}{\;V}^{1/2}$$

where I_p_ = peak current, A = electroactive surface area, cm^2^, *n* = electron stoichiometry, D = diffusion coefficient, cm^2^/s (i.e, 7.6 × 10^−6^ cm^2^/s according to the literature), C = concentration, mol/cm^3^, V = scan rate, volt/s.

The active surface area value was calculated for each electrode, and found to be 0.388 cm^2^ for the CNF-IL/PGE, 0.379 cm^2^ for the IL/PGE, 0.247 cm^2^ for CNF/PGE and 0.21 cm^2^ for the unmodified PGE (Table S2). In comparison to the surface area values, it is clearly seen that the electroactive surface area increased most significantly after the modification of the electrode surface with CNF-IL.

The electrochemical characterization by electrochemical impedance spectroscopy (EIS) was investigated before and after CNF-IL modification. Rct values measured by EIS are given in Table S3. While the average Rct value was 56.68 ± 19.76 Ohm with PGE, 208.40 ± 33.53 Ohm with PGE (control), 62.05 ± 19.80 Ohm with IL-PGE, 159.00 ± 41.32 Ohm for CNF-PGE and 53.22 ± 3.63 Ohm for CNF-IL/PGE. After the composite modification to the electrode surface, a decrease in the Rct value was observed (Figure 3). According to the data, 208.40 ± 33.53 Ohm resistance was measured with PGE (control) and 53.22 ± 3.63 Ohm with CNF-IL/PGE. The 74.46% decrease observed in the Rct value in the presence of CNF-IL composite modification can be explained by the increase in the conductivity of the aptasensor as a result of increasing electron transfer between the CNF-IL composite and the electrode interface.

According to the equation described by Janek et al. [76], the apparent fractional coverage ($\theta_{\mathrm{IS}\;}^{R}$) was calculated. The apparent fractional coverage value ($\theta_{\mathrm{IS}\;}^{R}$) for PGE and CNF-IL/PGE was calculated according to Equation (2);

(2) $$\theta_{\mathrm{IS}\;}^{R}=1-\frac{R_{\mathrm{CT}}^{a}}{R_{\mathrm{CT}}^{b}}$$

where $R_{\mathrm{CT}}^{a}$ = the charge transfer resistance before DNA-Apt immobilization, $R_{\mathrm{CT}}^{b}$ = the charge transfer resistance after DNA-Apt immobilization. The increase in Rct value was obtained by using both electrodes; PGE and CNF-IL/PGE after 0.2 µg/mL DNA-APT immobilization onto the surfaces of these electrodes. Due to the increase in repulsion force between the negatively charged DNA-Apt and the anionic redox probe, there was an increase in Rct obtained similarly to the earlier studies [8,29,63]. However, it was observed that the increase in Rct value by CNF-IL/PGE was found higher than the one by PGE in the presence of DNA-Apt immobilization (Figure S3). Accordingly, the higher apparent fractional coverage ($\theta_{\mathrm{IS}\;}^{R}$) was recorded as 0.91 by CNF-IL/PGE in comparison to the one (i.e, 0.83) obtained by PGE.

### 3.3. The Optimization of DNA-Apt Concentration

The interaction of aptamer prepared in PBS and APC prepared in PBS or TBS was examined (Table S4). According to the results obtained before and after 0.05 µg/mL APC-specific DNA-Apt:0.2 µg/mL APC (1:4) interaction, the highest percentage of increase (58%) was observed in the presence of interaction of APC prepared in PBS medium with DNA-Apt. Therefore, it was decided to continue the experiments with APC prepared in a PBS medium.

**Figure 4:**
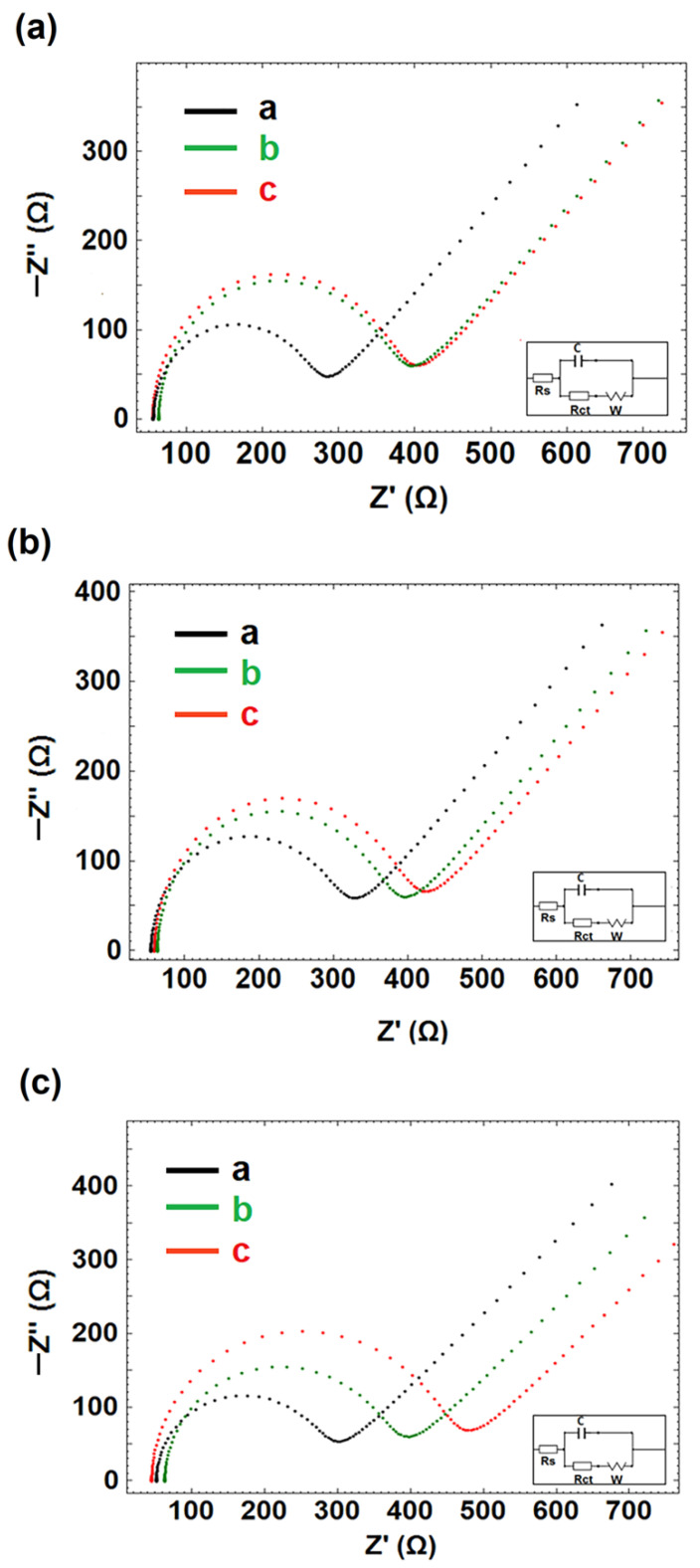
In the presence and absence of 0.2 µg/mL APC with (**a**) 0.005 µg/mL DNA-Apt, (**b**) 0.01 µg/mL DNA-Apt (**c**) 0.1 µg/mL DNA-Apt, the Niquist curves obtained in redox solution: (a) DNA-Apt/CNF-IL/PGE, (b) APC/CNF-IL/PGE, (c) APC/DNA-Apt/CNF-IL/PGE.

Since approximately 0.2 µg/mL APC was used in the study of Hamedani et al. [47], the optimization studies were performed at this concentration level of APC. The Rct value was measured before and after interaction with APC in various concentrations of DNA-Apt; 0.005, 0.01 and 0.1 µg/mL (shown in Figure 4). According to the measured Rct values, the highest increase of 47.43% was observed in the presence of 0.1 µg/mL DNA-Apt:0.2 µg/mL APC (1:2 ratio) (334.67 ± 54.37 Ohm (RSD% = 16.25%, *n* = 3). A total of 0.1 µg/mL DNA-Apt was chosen as the optimum concentration in our study. Next, the immobilization time study of 10 and 20 min was performed at a concentration of 0.1 µg/mL DNA-Apt and 0.6 µg/mL APC. According to the Rct values obtained (290.83 ± 33.03 Ohm, RSD% = 11.36%), the most reproducible results were obtained in immobilization time of 10 min, therefore it was chosen as the optimum interaction time (Figure S4).

In our present study, the interaction between APC-specific DNA aptamer and APC protein was investigated in the solution phase. The DNA aptamer and APC protein were allowed to interact in the solution phase for 5 min, followed by immobilization on the electrode surface for 10 min, after which impedimetric measurements were carried out. As the analysis process is not so long, there is no risk in terms of the stability of the sensor. Similarly, the developed CNF/IL-modified single-use electrodes were used just after their preparation procedure including 30 min of modification time and 10 min of drying. The total analysis time of APC by using an impedimetric-based electrochemical aptasensor showed results within 55 min. For this reason, there is no need to explore the stability of single-use aptasensor since it is prepared freshly and accordingly applied for the detection of APC.

### 3.4. The Optimization of APC Concentration in Buffer Medium

**Figure 5:**
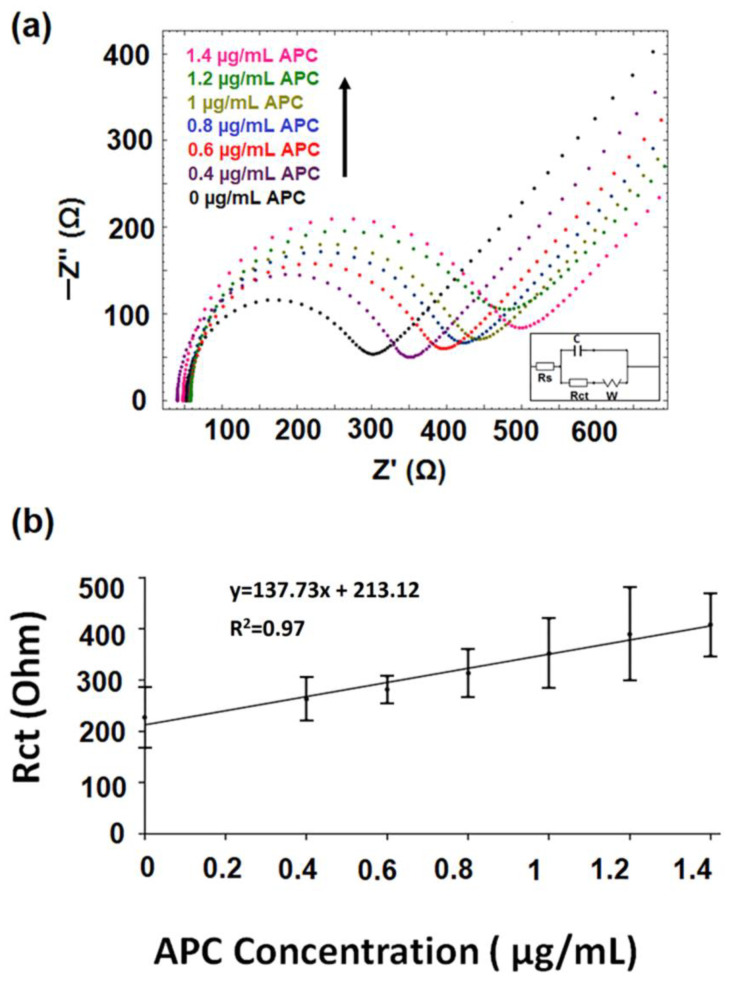
In the presence of 0.1 µg/mL DNA-Apt, (**a**) Nyquist diagrams were obtained in the absence/presence of APC in its increasing concentrations in buffer medium (**b**) Calibration curve for impedimetric determination of APC in buffer medium.

In the study of Hamedani et al. [47], the interaction of APC with immobilized HS02-52G aptamers was investigated over a wide range of APC concentrations between 31 and 500 nM. Since it is aimed herein to develop a more sensitive assay for APC detection by using CNF-IL/PGE in combination with the EIS technique, we tested the applicability of our assay in the lower concentration range of APC than 30 nM (equal to 1.8 μg/mL APC). Hence, the interaction of DNA-Apt with APC was studied in the present study in various concentrations of APC from 0.4 to1.6 µg/mL (shown in Figure S5). The measurement by the EIS technique was recorded before and after the interaction of DNA-Apt with APC in a buffer medium. There was an increase in Rct value while increasing APC concentration due to the fact that aptamers interact with more APC molecules, which have a negative structure in the redox probe (pH 5.93) [77]. Accordingly, the linearity of the response was recorded in the range of 0.4–1.4 µg/mL APC (Figure 5). The calibration graph with the regression equation Rct = 137.73C_APC_ + 213.12 with 97% confidence was presented in Figure 5B and the data was given in Table S5. According to the Miller and Miller method [54], the limit of detection (LOD) for APC was estimated as 0.23 µg/mL (equal to 3.83 nM). The sensitivity of the APC aptasensor was calculated and found to be 354.97 Ohm^.^mL/µg^.^cm^2^.

Since the most reproducible results (281 ± 26.39 Ohm and RSD% = 9.38%, *n* = 3) were obtained in an APC concentration of 0.6 µg/mL, it was selected herein as the optimum APC concentration in which the impedimetric detection of interaction of aptamer and APC was also explored in the presence of antisense oligonucleotide, AD22.

### 3.5. The Optimization of AD22 Concentration

The antisense oligonucleotide, AD22 reported by Hamedani et al. [47] is an oligonucleotide containing 22 bases that is sequence specific to DNA-Apt. AD22 matches with the 27 bases of aptamer (i.e, HS02-52G and also defined as DNA-Apt in the present work). Hamedani et al. [47] introduced HS02-52G as a highly specific APC inhibitor and they reported that the aptamer retains its high inhibitory activity in plasma and whole blood. Moreover, they presented that the functional activity of DNA aptamer within these matrices could be effectively reversed by the antisense molecule, AD22. Moreover, it was proposed by Hamedani et al. [47] that this aptamer–antidote pair could be a possible treatment option for acute APC-related bleeding complications. Under this scope, the effect of AD22 upon the interaction of DNA-Apt with APC was explored herein impedimetrically by using CNF-IL-PGE.

Firstly, the effect of AD22 concentration on the aptasensor response was investigated at different concentrations of AD22 such as; 0.005, 0.01 and 0.2 µg/mL in the presence of interaction between 0.1 µg/mL DNA-Apt and AD22. According to the change in Rct values (Table 1), an increase of about 15.09% was observed at Rct value (366 ± 71.19 Ohm and RSD% = 19.51%, *n* = 3) measured at 0.1 µg/mL AD22 concentration in comparison to the control group. As we expected, there should be the formation of a double helix form between DNA-Apt and AD22 while increasing the negativity with sugar-phosphate backbones [6,78]. Due to the increase in Rct value being observed only at 0.1 µg/mL AD22 concentration for its interaction of DNA-Apt, it was decided that the AD22 concentration should be kept as constant as 0.1 µg/mL in our further study.

Next, the effect of AD22 concentration on the aptasensor response was examined in the presence of 0.1 µg/mL DNA-Apt, 0.1 µg/mL AD22, and also 0.6 µg/mL APC. The Rct values measured before and after interaction DNA-Apt-AD22 with APC were shown in Table S7. After the interaction of DNA-Apt-AD22 with APC, the average Rct value was measured as 287.50 ± 17.31 Ohm (RSD% = 6.02% *n* = 3). In comparison to the average Rct value (366 ± 71.19 Ohm and RSD% = 19.51%, *n* = 3) measured in the presence of DNA Apt-AD22, a decrease (21.44%) was observed at the Rct measured in presence of the interaction of DNA-Apt-AD22 with APC.

### 3.6. The Optimization of Interaction Time of Aptamer-AD22 with Protein, APC

The interaction of AD22-DNA-Apt with APC was performed in different interaction times; 1, 2, and 5 min and the results were shown in Figure S6. After the interaction of AD22 with DNA aptamer, there is the formation of a double helix structure between DNA-Apt and its conjugate, AD22. After the immobilization of DNA-Apt-AD22 onto the electrode surface, a higher Rct value was recorded while increasing negativity at the electrode surface as a result of the double helix structure of DNA-Apt-AD22 base pair in contrast to DNA-aptamer alone, or AD22 control itself. In the 0.1 µg/mL DNA-Apt control group, the average Rct was measured as 201.33 ± 18.15 Ohm (RSD% = 9.01%, *n* = 3). As a result of the interaction between 0.1 µg/mL AD22 and 0.1 µg/mL DNA-Apt, it was obtained as 344.67 ± 39.17 Ohm (RSD% = 11.36%, *n* = 3) and hence, 82.64% increase was recorded as we expected (Figure S6A).

In the case of interaction of 0.1 µg/mL AD22, 0.1 µg/mL DNA-Apt with 0.6 µg/mL APC in 1 min interaction time, the average Rct value was recorded as 292 ± 55.76 Ohm (RSD% = 19.10%, *n* = 3). Considering the data recorded in the case of the interaction of DNA-Apt-AD22 with APC (Figure S6);

- In the presence of interaction between AD22 and DNA-Apt, a decrease of 15.28% was observed in contrast to the mean Rct value (344.67 ± 39.17 Ohm and RSD% = 11.36%, *n* = 3). Different APC interaction times (i.e, 2 and 5 min), the change in response resulted in a decrease of 0.29% and an increase of 18.76%, respectively.
- In the presence of interaction between DNA-Apt and APC, a decrease of 6.80% was observed in contrast to the mean Rct value (313.13 ± 96.76 Ohm and RSD% = 30.87%, *n* = 3). Different APC interaction times (i.e., 2 and 5 min), the change in response resulted in an increase of 26.50% and an increase of 52.54%, respectively.

While the average Rct was measured as 255 ± 19.97 Ohm (RSD% = 7.83%, *n* = 3) in the 0.6 µg/mL APC control group, it was recorded as 271 ± 108.19 Ohm (RSD% = 39.85%, *n* = 3) in the presence of interaction between 0.1 µg/mL AD22 and 0.6 µg/mL APC. Additionally, the average Rct value (271.50 ± 108.19 Ohm) measured in the interaction of APC with AD22 was found lower than the one (313.13 ± 96.76 Ohm) measured in the presence of interaction of DNA Aptamer with APC. Thus, an increase in the Rct value of approximately 6% was observed in contrast to the APC control group. Based on this data, it could be concluded that AD22 does not specifically bind to the target protein APC, which was also previously reported by Hamedani et al. [47].

### 3.7. The Applicability of APC Aptasensor in Artificial Serum Medium

The serum can be defined as a complex biological fluid that contains various compositions, such as proteins (albumins, globulins, etc.) and other biomolecules [68]. A majority of biosensors have presented some promising properties in aqueous buffers through monitoring of a specific biomolecule. However, regardless of this fact, the detection may be getting more difficult when it is applied in a serum media rather thanin an aqueous medium since the serum contains a large number of proteins or other biomolecules as a potential interferent. Therefore, the electrochemical performance of our impedimetric aptasensor was tested herein in the artificial serum; fetal bovine serum (FBS).

Further experiments were performed using fetal bovine serum (FBS) diluted 1:500, 1:1000, and 1:2000 with PBS (pH 7.40). The lowest and most reproducible Rct value (279 ± 33.15 Ohm and RSD% = 11.88%, *n* = 3) was observed in the medium of 1:2000 FBS (Figure S7B(c)). In the case of interaction of 0.1 µg/mL DNA-Apt with 0.6 µg/mL APC in the medium of 1:2000 FBS, a 15% increase was obtained in contrast to the Rct value measured in the presence of DNA-Apt alone (Figure S7C(a–c)). According to the data, we decided to perform further experiments in this medium; 1:2000 diluted FBS.

**Figure 6:**
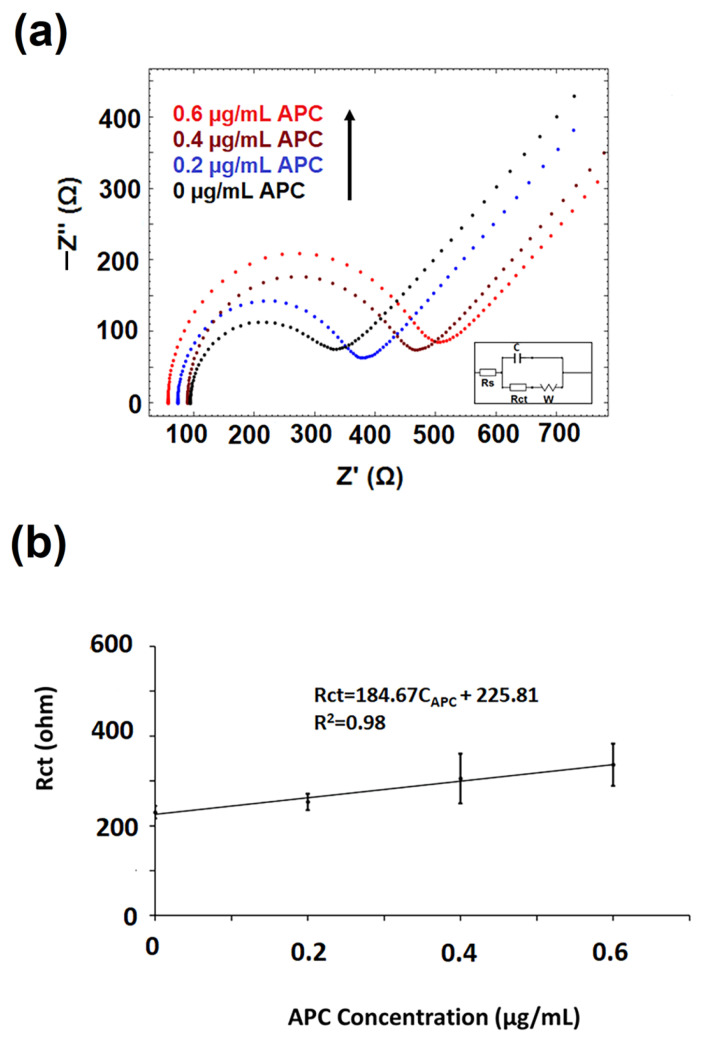
(**a**) Nyquist diagrams representing the response of aptasensor in different concentrations of APC in the medium of 1:2000 diluted FBS. (**b**) Calibration curve presenting the data for impedimetric determination of APC in 1:2000 diluted FBS.

After the interaction of 0.1 µg/mL DNA-Apt with APC in an FBS medium in the concentration range of 0.2–0.6 µg/mL APC, an increase in the Rct value was observed linearly (Figure 6B and Table S6). The resulting calibration graph based on the Rct values measured in the range of 0–0.6 µg/mL APC concentration in FBS medium was shown in Figure 6B and accordingly, the regression equation Rct = 184.67C_APC_ + 225.81 with 98% confidence was obtained. Accordingly, the limit of detection (LOD) [54] for APC in the medium of 1:2000 diluted FBS was calculated and found to be 0.11 µg/mL (equal to 1.83 nM) and the sensitivity was estimated as 475.953 Ohm·mL/µg·cm^2^.

### 3.8. Selectivity Studies

**Figure 7:**
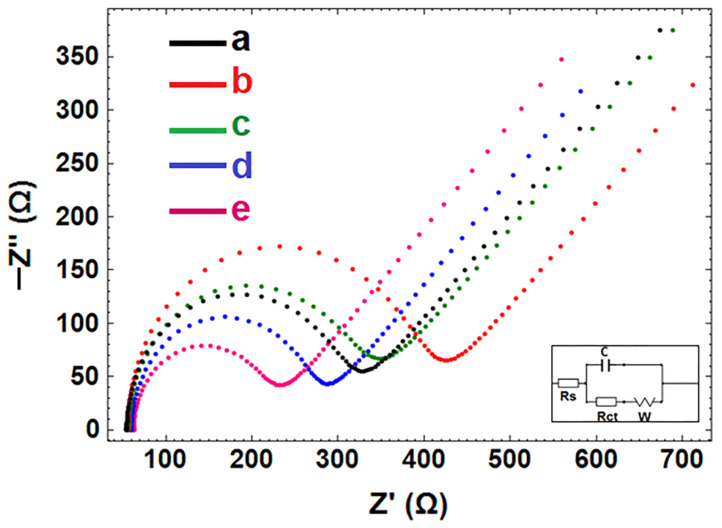
Nyquist diagrams recorded after (a) 0.1 µg/mL DNA-Apt immobilization onto CNF-IL modified electrode and aptamer interaction with 0.6 µg/mL of (b) APC, (c) PC, (d) THR, (e) BSA in 1:2000 diluted FBS.

In selectivity experiments, Rct values were measured in the case of interaction between 0.1 µg/mL DNA-Apt and 0.6 µg/mL of each protein; APC, PC, THR and BSA. The results were shown in Figure 7 and Table S8. After the interaction of aptamer with proteins, the average Rct values were measured as 332.50 ± 14.27 Ohm (RSD% = 4.29%, *n* = 3), 257.33 ± 29.54 Ohm (RSD% = 11.4%, *n* = 3), 202.00 ± 33.60 Ohm (RSD% = 16.63%, *n* = 3), 157 ± 9.19 Ohm (RSD% = 5.84%, *n* = 3) respectively, in the presence of APC, PC, THR and BSA. The results show that the APC-specific DNA aptamer can recognize and interact with its target protein with high selectivity in comparison to other proteins even in a medium such as FBS. Since APC, PC, THR and FVa work together in the PC anticoagulant pathway, APC has different binding active sites for each of these proteins, PC, FVa and THR [79]. In the case of the interaction of APC with aptamer as well as the one with PC, there was an increase recorded at Rct value of 39.70% and 8.12%, respectively. However, a decrease in Rct was observed in the presence of aptamer interaction with THR or BSA respectively of 15.2% and 33.82% (shown in Table S8).

### 3.9. Impedimetric Detection of Interaction of Aptamer-AD22 with APC in FBS

**Figure 8:**
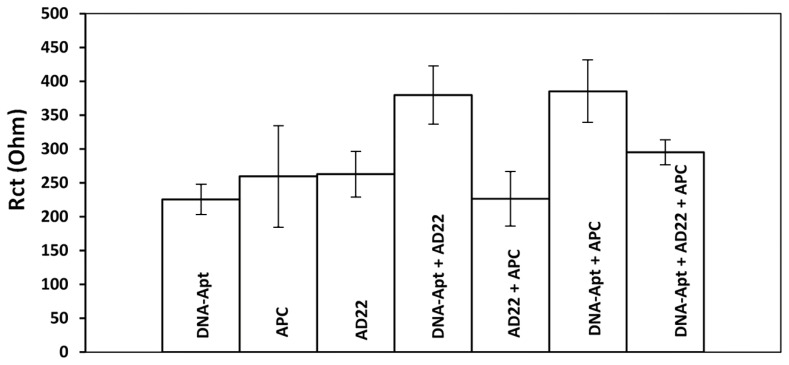
Histograms representing the average Rct values (*n* = 3) obtained by the interaction of 0.1 µg/mL DNA-Apt and 0.6 µg/mL APC in the absence and presence of 0.1 µg/mL AD22 in 1:2000 diluted FBS.

The interaction of antisense oligonucleotide, AD22 with aptamer and their interaction with APC in the FBS medium was explored under the conditions of 0.1 µg/mL DNA-Apt, 0.6 µg/mL APC and 0.1 µg/mL AD22. Then, EIS measurements were performed. The results obtained are shown in Figure 8 and Table S9.

The Rct values measured before and after interaction with DNA-Apt-AD22 with APC were shown in Table S9 in the presence and absence of AD22. Similar to the results previously reported in the literature [60,61,62,63], the Rct value was measured after the interaction between aptamer and APC in buffer and also in artificial serum resulting in a higher increase of 54.45% and 70.84%, respectively in comparison to the Rct value obtained in the presence of DNA-Apt (Figure 8 and Table S9). In contrast to the Rct value (385.25 ± 46.02 Ohm and RSD% = 11.94%, *n* = 3) measured in the presence of interaction of DNA-Apt with APC, a 23.23% decrease was obtained at Rct value measured in the presence of DNA-Apt-AD22 interaction with APC (i.e, 295.75 ± 18.41 Ohm (RSD% = 6.24%, *n* = 3).

## 4. Conclusions

The development of a solution phase aptasensor specific to APC and its application to impedimetric detection of APC was explored by using CNF-IL composite modified electrodes CNF-IL/PGE. The experimental parameters for the solution-phase interaction of APC and APC-specific DNA aptamer as well as APC inhibitor; aptamer–antidote pair was optimized, such as APC concentrations, and interaction time. The applicability of the impedimetric aptasensor for APC detection was also explored in fetal bovine serum, FBS as well as its selectivity over numerous proteins like protein C, thrombin, bovine serum albumin.

Within the scope of the study, the development of disposable electrodes modified with composite nanomaterials containing CNF and ionic liquid allowed us to develop an APC-specific impedimetric aptasensor, which resulted in APC detection in high sensitivity with good selectivity. The detection limit for APC was found to be 0.23 µg/mL (equal to 3.83 nM) in buffer as well as 0.11 µg/mL (equal to 1.83 nM) in artificial serum. The sensitivity was calculated and found to be 354.97 Ohm.mL/µg.cm^2^ and 475.953 Ohm.mL/µg.cm^2^ in buffer and artificial serum medium, respectively.

Existing methods for APC detection have some disadvantages while requiring specialized personnel, costly equipment, and a long assay time. In comparison to earlier studies in the literature [51,52,53,54,55,56,57], our assay provides a faster and more cost-effective protocol for reliable, sensitive and selective analysis of APC. This assay offers some advantages over previous studies on APC detection (shown in Table 2) in terms of short assay time (i.e, 55 min), ease of application, and low cost per analysis. In comparison to previous reports on carbon nanofiber-based aptasensor technology, impedimetric aptasensors presented herein an efficient protocol requiring a lower sample amount as well as faster detection of protein. In addition, our aptasensor was developed as a disposable, cost-effective protocol that requires fewer chemicals in comparison to other conventional methods like ELISA [51,52,53,54,55,56,57].

The aptasensor we developed enables the detection of APC in a relatively short time (i.e., 55 min), making our assay a practical method compared to other conventional methods for APC detection in the literature. In comparison to previous reports on APC detection, our impedimetric aptasensor array system presented an efficient protocol requiring lower performance. Additionally, our developed impedimetric-based aptasensor is not only disposable but also very cost-effective since it is based on a pencil graphite electrode. Hence EIS technique based on single-use electrodes provides a time-saving protocol requiring fewer chemicals than other conventional methods based on ELISA reported in the literature [51,52,53,54,55,56,57], but also selectively determined APC in comparison to other biomolecules such as PC, THR, and BSA.

Aptasensors specific to APC were presented previously with their applications in the medium of artificial serum [60,61,63,65]. In the present study, we obtained a lower detection limit for APC in comparison to earlier studies [60,61,63]. In addition, this aptasensor was tested in the artificial serum medium and accordingly, it resulted in a lower LOD in comparison to the one in the buffer medium. The impedimetric aptasensor developed for APC detection has several advantages; such as resulting in a short assay time, easy application, low cost per analysis, and implementation of the POC system. In addition, the sensor is developed as single-use while eliminating the need for complex cleaning and maintenance procedures. However, further validation studies are necessary to ensure the accuracy and reliability of the sensor under the clinical settings for ASSURED-compliant point-of-care diagnostics.

The aptasensor developed herein will not only expand the applications of novel carbon nanomaterial-based composites, but also we believe that this electrochemical aptasensing approach will open a new path for the expansion of novel aptasensors as well as the monitoring of the interaction of aptamers with their target analytes (proteins, toxins, etc.) in the absence/presence of aptamer–antisense pair.

## Figures and Tables

**Table 1:** The results obtained in the absence/presence of interaction DNA-Apt in different concentrations of AD22 with average Rct values (*n* = 3) and RSD%.

Experimental Group	Average Rct Values Measured Only in the Presence of AD22	Average Rct Value Measured with 0.1 µg/mL DNA-Apt in the Presence of AD22	RSD%	Change in the Rct Value
0.1 µg/mL DNA-Apt (control grup)	213.33 ± 14.15	-	6.63	6.63 fold increase *
0.05 µg/mL AD22	317.50 ± 82.73	206.00 ± 22.63	10.98	35.12% decrease
0.1 µg/mL AD22	318.00 ± 30.14	366.00 ± 71.39	19.51	15.09% increase
0.2 µg/mL AD22	345.00 ± 92.93	293.33 ± 64.94	22.14	14.98% decrease

* 6.63 fold increase was recorded at Rct measured only after immobilization of DNA-Apt onto the surface of CNF-IL/PGE.

**Table 2:** Overview of some of the previous methods developed using DNA aptamers for detecting activated protein C.

Method	Detection Limit	Reference
SPR	1.5 ng/mL	[28]
EIS	0.74 µg/mL in buffer	[60]
	2.03 µg/mL in FBS	
EIS	1.81 µg/mL in buffer	[61]
	0.02 µg/mL in FBS	
DPV	2.35 µg/mL in buffer	[62]
EIS, CV	0.94 µg/mL in buffer	[63]
	1.5 µg/mL in FBS	
DPV	0.73 × 10^−5^ µg/mL	[65]
EIS	0.23 µg/mL in buffer	This work
	0.11 µg/mL in FBS	

EIS, electrochemical impedance spectroscopy; SPR, surface plasmon resonance; DPV, differential pulse voltammetry; CV, cyclic voltammetry.

## Data Availability

The data presented in this study are available within the article and its Supplementary Materials. Other data that support the findings of this study are available upon request from the corresponding author as well as co-author.

## Supplementary Materials

The following supporting information can be downloaded at: https://www.mdpi.com/article/10.3390/bios13040458/s1, Figure S1. The EDX spectrum on the surface (A) PGE (control), (B) CNF-PGE, (C) IL-PGE, (D) CNF-IL/PGE; Figure S2. The element concentrations for carbon (C), fluorine (F), nitrogen (N), oxygen (O), phosphorus (P), silicon (Si), atoms n the surface of (A) PGE (control), (B) CNF/PGE, (C) IL/PGE, (D) CNF-IL/PGE; Table S1. The RSD % value and Ia increase values in the mean anodic current measured in voltammetric examination of the effect of % IL change on the response with CV technique (n = 3); Table S2. The average values (n = 3) of the anodic peak current (Ia), the cathodic current (Ic), anodic charge value (Qa), the cathodic charge value (Qc) and the calculated surface area (A) obtained by unmodified PGE, PGE (control), CNF/PGE, IL/PGE, CNF-IL/PGE; Table S3. The average Rct values (n = 3) obtained by PGE, PGE (control), CNF/PGE, IL/PGE, CNF-IL/PGE electrodes; Figure S3. 0.2 µg /mL DNA-Apt before and after immobilization (A) (a) PGE, (b) DNA-Apt/PGE, (B) (a) CNF-IL/PGE, (d) DNA-Apt /CNF-IL/PGE, Niquist curves of measurements performed in redox solution containing 2.5 mM Fe(CN)6 ^3−/4−^; Table S4. Buffer effect after interaction of 0.05 µg/mL DNA-Apt and 0.2 µg/mL APC (n = 3); Figure S4. Niquist diagrams of the 10 and 20 min immobilization time study at 0.1 µg/mL DNA-Apt and 0.6 µg/mL APC concentration; Table S5. Average Rct value (n = 3) measured with DNA-Apt before/after interaction with APC in its increasing concentrations in buffer medium; Figure S5. The average Rct value measured before and after 0.1 µg/mL DNA-Apt interaction with APC in various concentration ranging from 0 to 1.6 µg/mL at CNF-IL/PGE surface; Table S6 Average Rct value (n = 3) measured with DNA-Apt before/after interaction with APC in its increasing concentrations in FBS medium; Figure S6. Histograms representing average Rct values (n = 3) obtained in the absence and presence of the interaction of 0.1 µg/mL DNA-Apt and 0.6 µg/mL APC with 0.1 µg/mL AD22 in its interaction time: (a) 1 min (b) 2 min (c) 5 min; Figure S7. Niquist diagrams obtained by CNF-IL modified PGE electrodes: (A) 0.1 µg/mL DNA-Apt modified electrode prepared (a) in PBS, (b) in FBS; (B) In different dilution ratio (FBS:PBS) (a) 1:500, (b) 1:1000, (c) 1:2000 and (C) In the dilution ratio (1:2000) of FBS (a) 0.1 µg/mL DNA-Apt, (b) 0.6 µg/mL APC, (c) Interaction occured between 0.1 µg/mL DNA-Apt and 0.6 µg/mL APC; Table S7. The average Rct values (n = 3) measured before and after interaction of 0.1 µg/mL DNA-Apt with 0.6 µg/mL APC in the presence of 0.1 µg/mL AD22; Table S8. The average Rct value (n = 3) measured before/after interaction of 0.1 µg/mL DNA-Apt with 0.6 µg/mL APC, PC, THR, BSA in serum medium diluted 1:2000 FBS:PBS; Table S9. The average Rct values (n = 3) measured before and after interaction of 0.1 µg/mL DNA-Apt with 0.6 µg/mL APC in the presence of 0.1 µg/mL AD22 in artificial serum medium.
