# Supplementary material for: Carbon Nanofiber-Ionic Liquid Nanocomposite Modified Aptasensors Developed for Electrochemical Investigation of Interaction of Aptamer/Aptamer–Antisense Pair with Activated Protein C
Source: Biosensors (Basel). 2023 Apr 4;13(4):458. doi: 10.3390/bios13040458 (PMC10136435; doi:10.3390/bios13040458)
Supplement: Supplementary file 1 [file biosensors-13-00458-s001.zip › biosensors-2122393-supplementary.pdf]

Supplementary Material

Carbon nanofiber-ionic liquid nanocomposite modified aptasensors developed for electrochemical investigation of interaction of aptamer / aptamer-antisense pair with activated protein C

Meltem Maral [1,2] and Arzum Erdem [1,2] *

[1] Department of Material Science and Engineering, The Institute of Natural and Applied Sciences, Ege University, Bornova, 35100, Izmir, TURKEY

[2] Analytical Chemistry Department, Faculty of Pharmacy, Ege University, Bornova, 35100, Izmir, TURKEY

*** Corresponding author (A. Erdem) arzum.erdem@ege.edu.tr and arzume@hotmail.com**

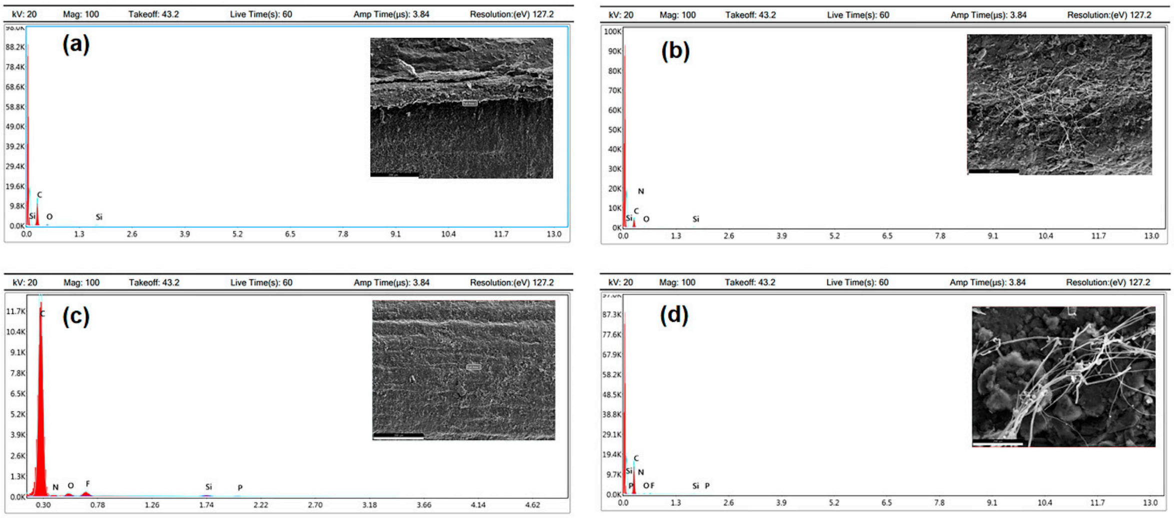

Figure S1. The EDX spectrum on the surface (A) PGE (control), (B) CNF-PGE, (C) IL-PGE, (D) CNF-IL/PGE.

(a)

Element	Weight %	Atomic %	Error %
C K	87.74	90.72	3.12
O K	11.54	8.96	12.19
SiK	0.71	0.32	6.33

(b)

Element	Weight %	Atomic %	Error %
C K	85.51	88.74	3.83
N K	4.31	3.83	28.15
O K	8.68	6.76	14.57
SiK	1.5	0.67	5.92

(c)

Element	Weight %	Atomic %	Error %
C K	83.65	86.93	3.04
N K	7.63	6.8	19.76
O K	5.09	3.97	14.37
F K	3.27	2.15	12.42
SiK	0.25	0.11	11.5
P K	0.12	0.05	23.29

(d)

Element	Weight %	Atomic %	Error %
C K	84.56	87.65	2.92
N K	7.23	6.42	21.74
O K	5.01	3.9	14.53
F K	2.89	1.89	12.54
SiK	0.24	0.1	11.64
P K	0.08	0.03	30.35

Figure S2. The element concentrations for carbon (C), fluorine (F), nitrogen (N), oxygen (O), phosphorus (P), silicon (Si), atoms n the surface of (A) PGE (control), (B) CNF/PGE, (C) IL/PGE, (D) CNF-IL/PGE.

Since the composite modified electrodes prepared with 5 % ionic liquid have the highest current value, the highest surface area, and the best reproducibility, it was decided to be the optimum ionic liquid percentage (Table S1).

Table S1. The RSD % value and Ia increase values in the mean anodic current measured in voltammetric examination of the effect of % IL change on the response with CV technique (n=3).

**Electrodes**	**Ia (µA)**	**RSD %**	**% increase in value of Ia**
PGE (control)	71.77 ± 17.08	23.81	-
CNF – 2 % IL/PGE	96.90 ± 17.57	18.13	35.01
CNF – 3 % IL/PGE	120.28 ± 13.76	11.44	67.6
CNF – 5 % IL/PGE	128.46 ± 3.39	2.64	79
CNF – 7 % IL/PGE	123.09 ± 4.33	3.52	71.5

Table S2. The average values (n=3) of the anodic peak current (Ia), the cathodic current (Ic), anodic charge value (Qa), the cathodic charge value (Qc) and the calculated surface area (A) obtained by unmodified PGE, PGE (control), CNF/PGE, IL/PGE, CNF-IL/PGE.

Electrodes	Ia (μA)	Qa (mC)	A (cm^2)	Ic (μA)	Qc (mC)	A (cm^2)
PGE	62.66 ± 20.89	1.30 ± 0.26	0.189	80.06 ± 20.57	0.97 ± 0.23	0.242
PGE (control)	71.77 ± 17.09	1.41 ± 0.13	0.217	84.91 ± 16.62	1.07 ± 0.10	0.256
CNF/PGE	81.94 ± 16.04	1.48 ± 0.16	0.247	94.43 ± 15.47	1.12 ± 0.12	0.285
IL/PGE	125.58 ± 6.14	1.68 ± 0.13	0.379	114.71 ± 65.89	1.28 ± 0.08	0.346
CNF-IL/PGE	128.47 ± 3.39	1.74 ± 0.05	0.388	116.975 ± 26.27	1.35 ± 0.04	0.353

Table S3. The average Rct values (n=3) obtained by PGE, PGE (control), CNF/PGE, IL/PGE, CNF-IL/PGE electrodes.

Electrodes	Average Rct value (Ohm)	% Change in Rct value	RSD %
PGE	56.68 ± 19.76	-	34.86
PGE (control)	208.40 ± 33.53	267.71 increase	16.09
CNF/PGE	159.00 ± 41.32	23.70 decrease	25.99
IL/PGE	62.05 ± 19.80	70.23 decrease	31.91
CNF-IL/PGE	53.22 ± 3.63	74.46 decrease	6.82

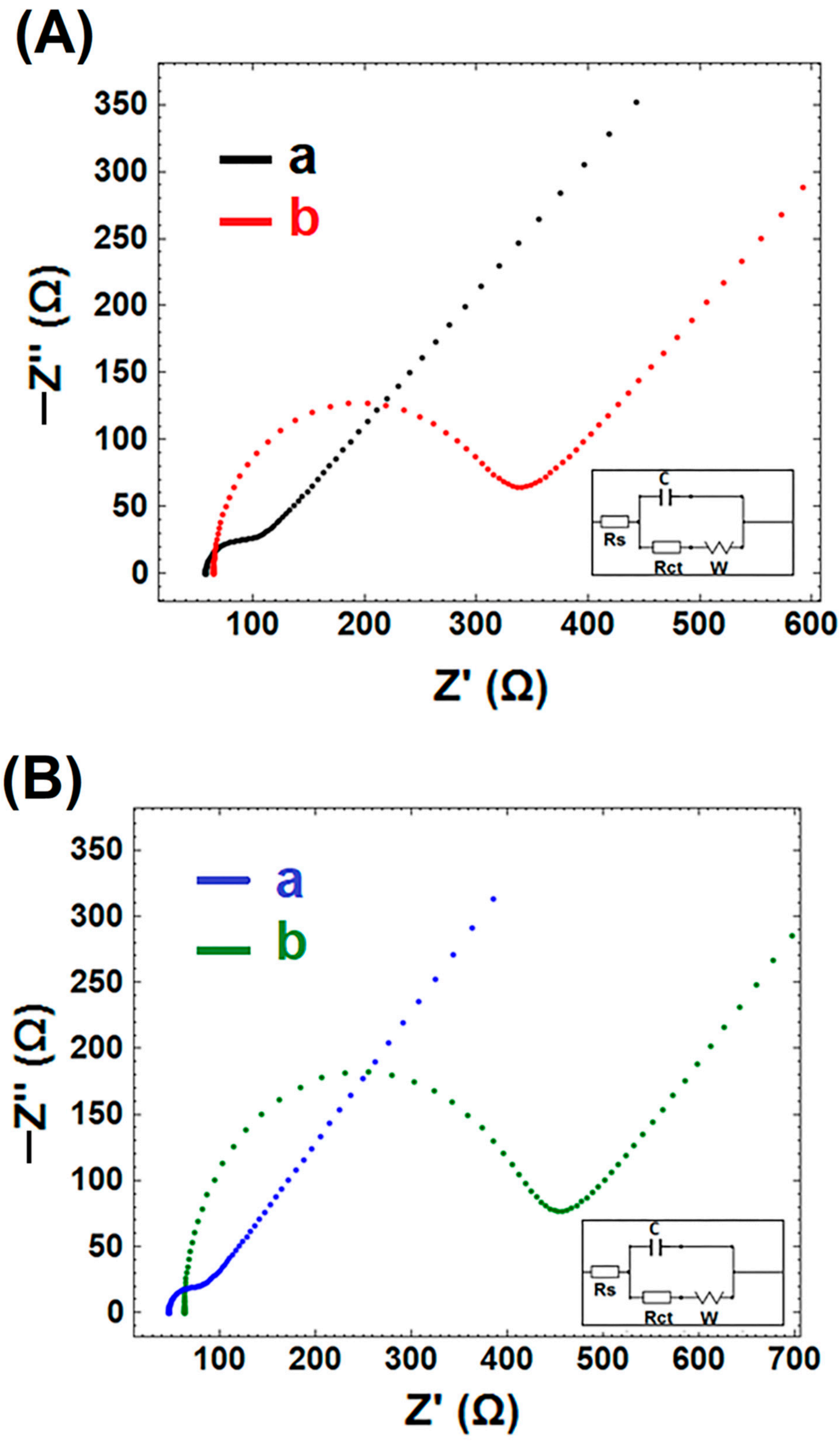

Figure S3. 0.2 µg /mL DNA-Apt before and after immobilization (A) (a) PGE, (b) DNA-Apt/PGE, (B) (a) CNF-IL/PGE, (d) DNA-Apt /CNF-IL/PGE, Niquist curves of measurements performed in redox solution containing 2.5 mM $Fe(CN)_6{}^{3-/4-}$.

Table S4. Buffer effect after interaction of 0.05 µg/mL DNA-Apt and 0.2 µg/mL APC (n=3).

Experimental group	Interaction experiment group with APC prepared in PBS medium	Interaction experiment group with APC prepared in TBS medium
	Average Rct value (Ohm)	
DNA-Apt (control experiment)	233.6 ± 42.18	
DNA-Apt : APC	371 ± 4 1.01	242.5 ± 62.93
APC (control experiment)	200.5 ± 85.56	209 ± 2 8.28
% change in Rct value relative to DNA-Apt	58 % Increase	3 % Increase

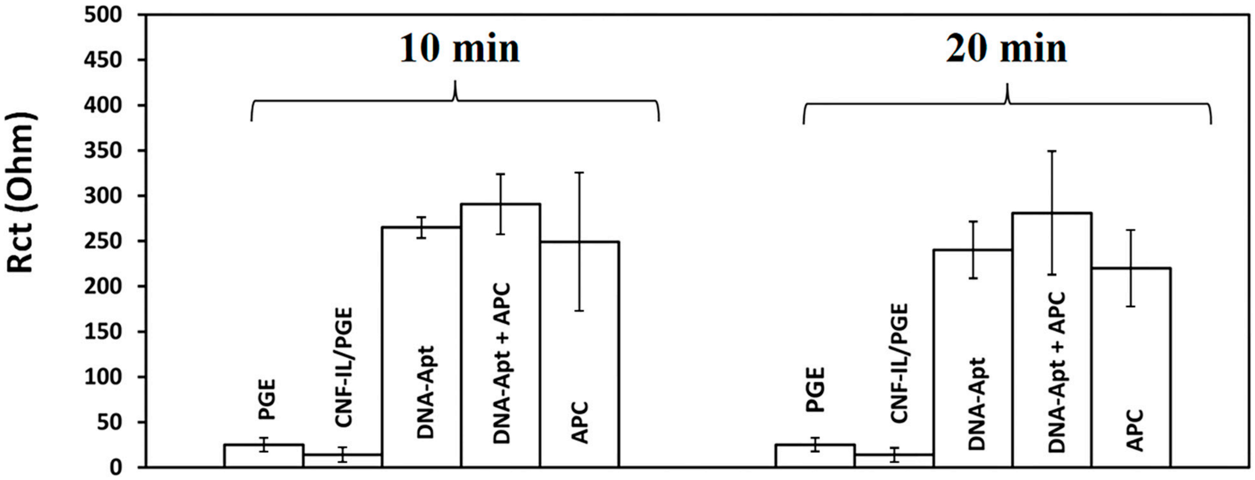

Figure S4. Niquist diagrams of the 10 and 20 min immobilization time study at 0.1 µg/mL DNA-Apt and 0.6 µg/mL APC concentration.

Table S5. Average Rct value (n=3) measured with DNA-Apt before/after interaction with APC in its increasing concentrations in buffer medium.

Experimental group	Average Rct value (Ohm)	RSD %	% change in Rct value relative to DNA-Apt
0.1 µg/mL DNA-Apt	227.00 ± 59.16	26,06	1.82 fold Increase*
0.1 µg/mL Apt + 0.4 µg/mL APC	263.25 ± 42.30	16.07	15.96 % Increase
0.1 µg/mL Apt + 0.6 µg/mL APC	281.40 ± 26.39	9.38	23.96 % Increase
0.1 µg/mL Apt + 0.8 µg/mL APC	313.40 ± 46.20	14.74	38. 06 % Increase
0.1 µg/mL Apt + 1 µg/mL APC	352.50 ± 67.45	19.14	55.28 % Increase
0.1 µg/mL Apt + 1.2 µg/mL APC	389.80 ± 90.16	23.13	71. 71 % Increase
0.1 µg/mL Apt + 1.4 µg/mL APC	408.20 ± 60.96	14.94	79.82 % Increase

*1.82 fold increase was observed in the mean Rct value measured in the presence of DNA-Apt immobilization to the CNF-IL/PGE surface.

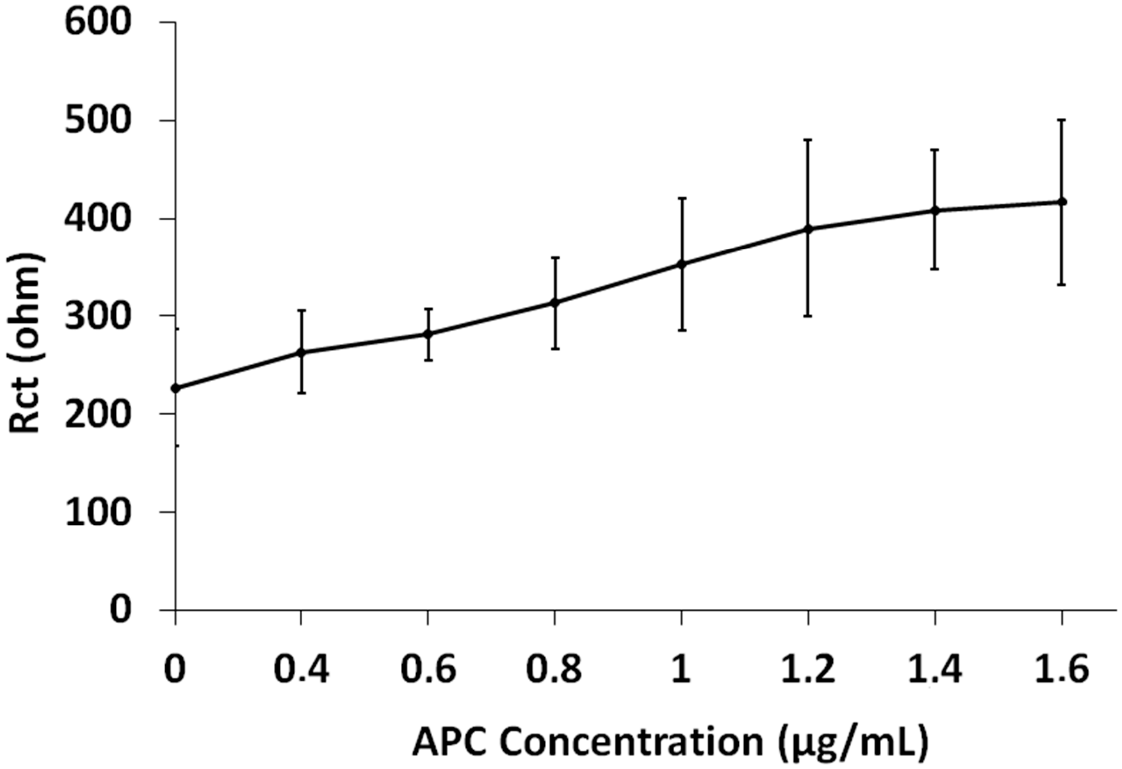

Figure S5. The average Rct value measured before and after 0.1 µg/mL DNA-Apt interaction with APC in various concentration ranging from 0 to 1.6 µg/mL at CNF-IL/PGE surface.

Table S6 Average Rct value (n=3) measured with DNA-Apt before/after interaction with APC in its increasing concentrations in FBS medium.

Experimental group	Average Rct value (Ohm)	RSD %	% change in Rct value relative to DNA-Apt
0.1 µg/mL DNA-Apt	230.25 ± 14.01	6.08	7.13 fold Increase*
0.1 µg/mL Apt + 0.2 µg/mL APC	253.25 ± 18.41	7.27	9.98 % Increase
0.1 µg/mL Apt + 0.4 µg/mL APC	305.33 ± 55.82	18.28	32.60 % Increase
0.1 µg/mL Apt + 0.6 µg/mL APC	336.00 ± 46.99	13.98	41.75 % Increase

*7.13 fold increase was observed in the mean Rct value measured in the presence of DNA-Apt immobilization to the CNF-IL/PGE surface.

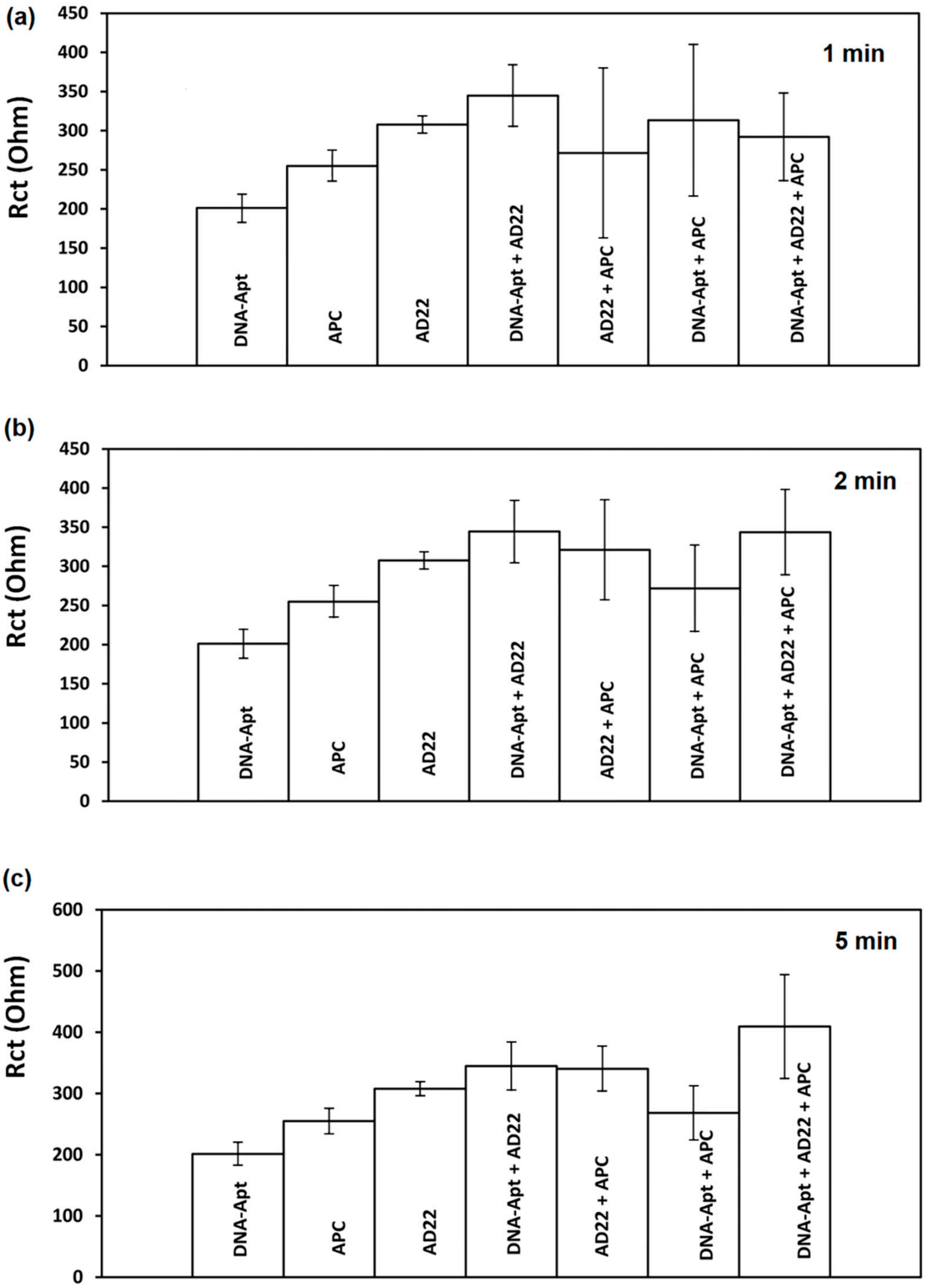

Figure S6. Histograms representing average Rct values (n=3) obtained in the absence and presence of the interaction of 0.1 µg/mL DNA-Apt and 0.6 µg/mL APC with 0.1 µg/mL AD22 in its interaction time: a) 1 minute b) 2 minutes c) 5 minutes.

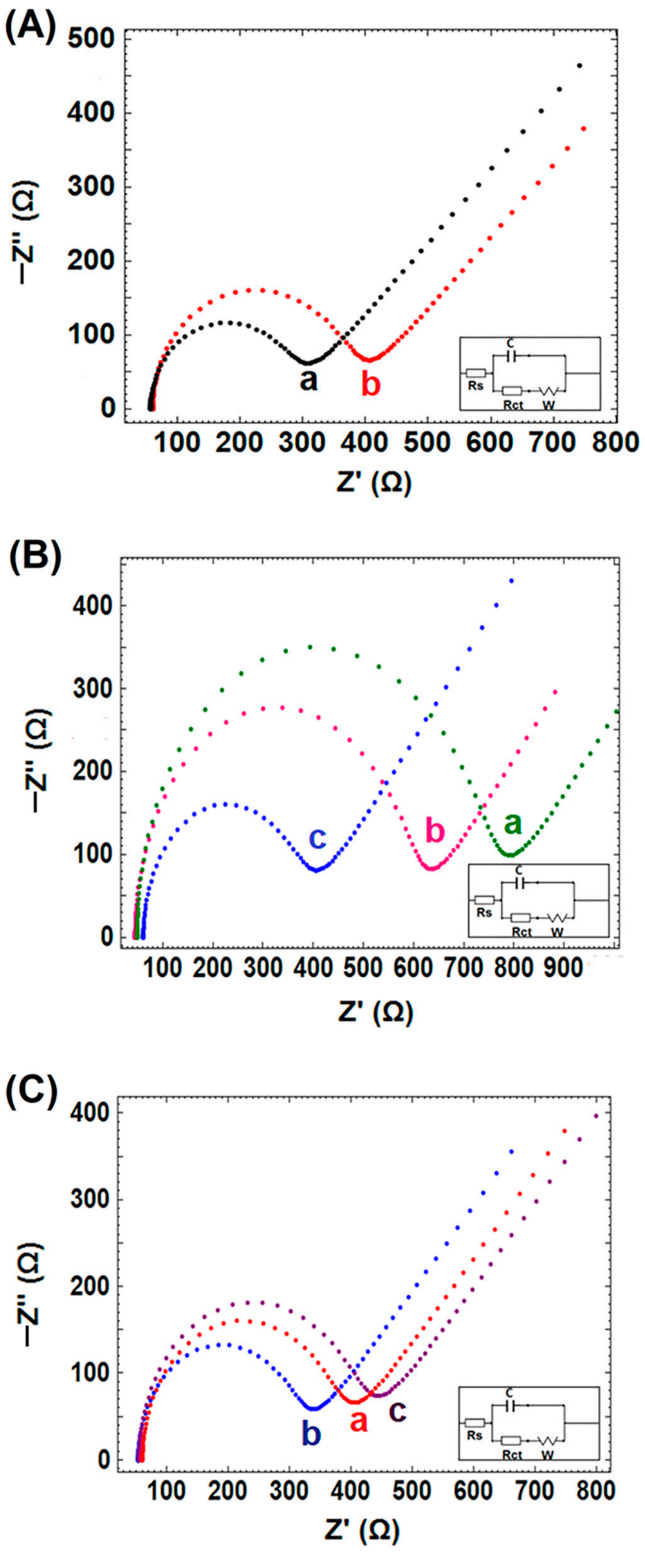

Figure S7. Niquist diagrams obtained by CNF-IL modified PGE electrodes: (A) 0.1 µg/mL DNA-Apt modified electrode prepared (a) in PBS, (b) in FBS; (B) In different dilution ratio (FBS : PBS) (a) 1 : 500, (b) 1 : 1000, (c) 1 : 2000 and (C) In the dilution ratio (1 : 2000) of FBS (a) 0.1 µg/mL DNA-Apt, (b) 0.6 µg/mL APC, (c) Interaction occured between 0.1 µg/mL DNA-Apt and 0.6 µg/mL APC.

Table S7. The average Rct values (n=3) measured before and after interaction of 0.1 µg/mL DNA-Apt with 0.6 µg/mL APC in the presence of 0.1 µg/mL AD22.

No	Experimental group	Average Rct value (Ohm)	RSD %	Change
1	DNA-Apt (control experiment)	213.33 ± 14.15	6.63	6.08 fold Increase*
2	AD22	318.00 ± 30.14	9.48	9.07 fold Increase*
3	DNA-Apt + AD22	366.00 ± 71.39	19.51	
4	DNA-Apt + APC	329.50 ± 48.37	14.68	
5	AD22 + APC	276.75 ± 89.41	32.31	
6	DNA-Apt + AD22 + APC	287.50 ± 17.31	6.02	
	% change in Rct values from the 1st experimental group to the 4th experimental group			54.45 % Increase
	% change in Rct values from the 2nd experimental group to the 5th experimental group			12.97 % decrease
	% change in Rct values from the 3rd experimental group to the 6th experimental group			21.44 % decrease
	% change in Rct values from the 4th experimental group to the 6th experimental group			12.75 % decrease
*	APC (control experiment)	248.25 ± 21.17<br>RSD %=8.53 %		7.08 fold Increase*

** The average Rct value measured after only DNA-Apt or AD22 or APC immobilization onto the CNF-IL/PGE surface, respectively; 6.08, 9.07 and 7.08 fold increase was observed.*

Table S8. The average Rct value (n=3) measured before/after interaction of 0.1 µg/mL DNA-Apt with 0.6 µg/mL APC, PC, THR, BSA in serum medium diluted 1:2000 FBS:PBS.

Experimental group	Average Rct value (Ohm)	RSD %	Change
DNA-Apt	238 ± 6.24	2.62	8.99 fold Increase*
APC	235.33 ± 19.60	8.33	8.89 fold Increase*
PC	241.60 ± 61.52	25.46	9.12 fold Increase*
THR	218.20 ± 57.95	26.56	8.24 fold Increase*
BSA	196.25 ± 37.40	19.06	7.41 fold Increase*
DNA-Apt + APC	332.50 ± 14.27	4.29	39.70 % Increase
DNA-Apt + PC	257.33 ± 29.54	11.48	8.12 % Increase
DNA-Apt + THR	202.00 ± 33.60	16.63	15.12 % decrease
DNA-Apt + BSA	157 ± 9.19	5.84	33.82 % decrease

** The average Rct value measured after only DNA-Apt or APC or other proteins (PC, THR, BSA) immobilization onto the CNF-IL/PGE surface, respectively; 8.99, 8.89, 9.12, 26.56 and 19.06 fold increases were observed.*

Table S9. The average Rct values (n=3) measured before and after interaction of 0.1 µg/mL DNA-Apt with 0.6 µg/mL APC in the presence of 0.1 µg/mL AD22 in artificial serum medium.

**No**	**Experimental group**	**Average Rct value (Ohm)**	**RSD %**	**Change**
1	DNA-Apt (control experiment)	225.50 ± 21.76	9.65	9.5 fold Increase*
2	AD22	262.75 ± 33.36	12.7	11.16 fold Increase*
3	DNA-Apt + AD22	379.75 ± 42.84	11.28	
4	DNA-Apt + APC	385.25 ± 46.02	11.94	
5	AD22 + APC	226.50 ± 39.79	17.57	
6	DNA-Apt + AD22 + APC	295.75 ± 18.41	6.24	
	% change in Rct values from the 1st experimental group to the 4th experimental group			70.84 % Increase
	% change in Rct values from the 2nd experimental group to the 5th experimental group			13.79 % decrease
	% change in Rct values from the 3rd experimental group to the 6th experimental group			22.25 % decrease
	% change in Rct values from the 4th experimental group to the 6th experimental group			23.36 % decrease
*	APC (control experiment)	259.75 ± 74.71<br>RSD %=28.76 %		11.03 fold Increase*

** The average Rct value measured after only DNA-Apt or AD22 or APC immobilization to the CNF-IL/PGE surface, respectively; 9.05, 11.16 and 11.03 fold increase was observed.*
